# Case 4/2017 - Double-Chambered Right Ventricle with Dextrocardia and Hypoxemia Due to Atrial Shunt in a 4-Year-Old Girl

**DOI:** 10.5935/abc.20170078

**Published:** 2017-06

**Authors:** Edmar Atik, José Fernando Cavalini

**Affiliations:** Instituto do Coração do Hospital das Clínicas da Faculdade de Medicina da Universidade de São Paulo, SP - Brazil

**Keywords:** Double Chambered Right Ventricle, Dextrocardia, Hypoxia

## Clinical data

A premature female twin (33-week gestation), weighing at birth 1935 g, remained hospitalized for one month due to the diagnosis of atrial septal defect (ASD) + ventricular septal defect (VSD) + persistence of ductus arteriosus (PDA). The patient gained less weight than the average children, but maintained full and similar activity, receiving furosemide and captopril, up to the age of 3 years, when her mother noticed cyanosis.

### Physical exam

Eupnea; mild cyanosis; normal pulses; weight, 11 kg; height, 89 cm; heart rate, 100 bpm; O_2_ saturation, 83%. The aorta was not palpable at the suprasternal notch. Her chest showed mild bulging and mild systolic thrusts on the right sternal border (RSB). The 1^st^ heart sound was more intense on the right midclavicular line (RMCL), and the 2^nd^ heart sound, on the RSB with greater radiation to the RMCL. A rough systolic ejection murmur (4/6) was audible on the upper RSB, and a mild regurgitation systolic murmur (4/6) was audible on the lower RSB. The liver was palpated 1 cm from the right costal margin.

### Complementary diagnostic tests

**Figure 1:**
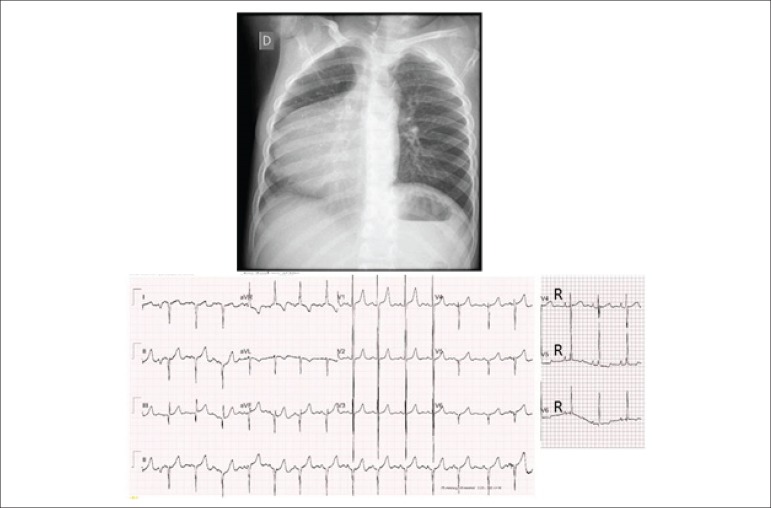
X-ray showing marked cardiomegaly with rounded and long ventricular arch to the right, situs solitus (gastric bubble to the left) and reduced pulmonary vascular bed. Electrocardiogram showing signs of marked right ventricular overload to the right, with preponderant R wave in V6R, S wave in V6, positive T wave in V6, and isoelectric T wave in V6R.

**Electrocardiogram:** sinus rhythm and signs of marked right ventricular overload. There were Rs complexes in V1 to V3, rsR´ in V5R and V6R, positive T wave in V1 to V6, and isoelectric T wave in V6R, signs of right ventricle (RV) located to the right. AP: +60º, AQRS: -150º, AT: +70º (Figure1).

**Chest X-ray:** enlargement of the cardiac silhouette to the right, and reduced pulmonary vascular bed. Rounded and long ventricular arch to the right (Figure1).

**Figure 2:**
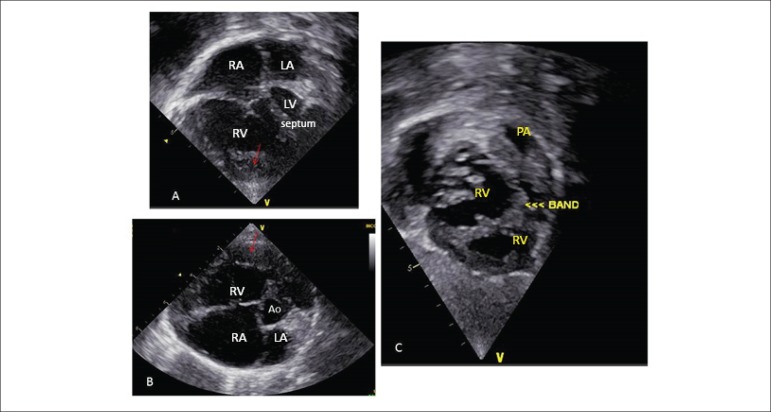
Echocardiogram: 4-chamber (A) and short-axis (B) views showing markedly enlarged right cardiac cavities with septa bulging to the left and marked ventricular hypertrophy (arrows), and moderator band dividing the two right ventricular chambers: proximal and distal chambers seen on subcostal view (C). RA: right atrium; LA: left atrium; Ao: aorta; RV: right ventricle; LV: left ventricle; PA: pulmonary artery.

**Echocardiogram:** (Figure2) showed situs solitus with dextrocardia, normal systemic and pulmonary venous connections, concordant atrioventricular and ventriculoarterial connections. Dilatation of the inferior vena cava and suprahepatic veins. Ostium secundum ASD of 4 mm, with right-to-left shunt. Intact ventricular septum deviated to the left. Marked tricuspid regurgitation. Aneurysmatic right atrium with volume of 58 mL/m^2^. Right ventricle markedly dilated and hypertrophied, with hypertrophied moderator band, narrow infundibulum due to hypertrophy, and two ventricular chambers with a 140-mmHg gradient between them. Normal pulmonary and aortic valves. Normal left cavities. PT = 20 mm, PA´s = 9 mm. Pulmonary ring = 15 mm and right ventricular anterior wall = 10 mm.

### Clinical diagnosis

Stenosis of double-chambered right ventricular inlet with mild hypoxia due to right-to-left shunt through a small ASD.

### Clinical rationale

The clinical elements were compatible with cyanotic congenital heart disease with reduced pulmonary flow resulting from an obstruction at the right and right-to-left shunt. An obstruction in the right ventricular inlet could be suspected based on the auscultation of a markedly rough and intense systolic murmur. However, the more intense 2^nd^ heart sound raised the possibility of corrected transposition of the great arteries, mainly in the presence of dextrocardia with situs solitus. The electrocardiogram was not compatible with atrioventricular discordance, because the T wave indicated a RV located to the right (T wave axis to the left (+70 degrees) and greater intensity in V6 than in V6R). The echocardiogram was conclusive about the defect and its repercussion. The marked tricuspid regurgitation causing an aneurysmatic right atrium was due to marked obstruction inside the RV. It is worth noting the rarity of that anomaly in the presence of dextrocardia with situs solitus and no VSD, in addition to marked tricuspid regurgitation as an uncommon consequence from obstruction in the RV.

### Differential diagnosis

The most likely differential diagnosis was corrected transposition of the great arteries.

### Management

Because of the marked repercussion of the defect, surgery was performed immediately, eliminating the obstruction of the inlet of the hypertrophied RV.

## Comments

The double-chambered RV or stenosis of the inlet of the RV is a rare congenital anomaly, in which an anomalous hypertrophied muscular band divides the RV into two cavities, the proximal being of high pressure, and the distal, of low pressure. Muscular obstruction develops over time, but rarely in adult age. The hypertrophied muscle is either the septoparietal or the septomarginal trabecula.

In over 95% of the cases, the stenosis of the inlet of the RV is associated with VSD, whose location determines the characteristic clinical findings. Thus, when the VSD is located before the obstruction, the clinical findings are similar to those of tetralogy of Fallot, and when the VSD is distal to the obstruction, those findings are similar to those of the VSD itself. It is worth noting that the grade of obstruction and the size of the VSD account for the magnitude of the findings.

To our knowledge, this is the first report on the association of double-chambered RV with dextrocardia and situs solitus and no VSD, whose clinical findings simulated those of marked pulmonary stenosis and consequent progressive tricuspid regurgitation.^1,2^
